# Modulation of Type-1 and Type-2 Cannabinoid Receptors by Saffron in a Rat Model of Retinal Neurodegeneration

**DOI:** 10.1371/journal.pone.0166827

**Published:** 2016-11-18

**Authors:** Rita Maccarone, Cinzia Rapino, Darin Zerti, Monia di Tommaso, Natalia Battista, Stefano Di Marco, Silvia Bisti, Mauro Maccarrone

**Affiliations:** 1 Biotechnology and Applied Clinical Sciences Department, University of L'Aquila, L’Aquila, Italy; 2 Faculty of Veterinary Medicine, University of Teramo, Teramo, Italy; 3 Faculty of Bioscience, University of Teramo, Teramo, Italy; 4 ARC Centre of Excellence in Vision Science, John Curtin School of Medical Research, Australian National University, Canberra ACT 0200, Australia; 5 Interuniversity Consortium “Istituto Nazionale Biostrutture e Biosistemi” (INBB), Rome, Italy; 6 Department of Medicine, Campus Bio-Medico University of Rome, Rome, Italy; 7 European Center for Brain Research, IRCCS Santa Lucia Foundation, Rome, Italy; Instituto Murciano de Investigacion y Desarrollo Agrario y Alimentario, SPAIN

## Abstract

Experimental studies demonstrated that saffron (*Crocus sativus*) given as a dietary supplement counteracts the effects of bright continuous light (BCL) exposure in the albino rat retina, preserving both morphology and function and probably acting as a regulator of programmed cell death [1]. The purpose of this study was to ascertain whether the neuroprotective effect of saffron on rat retina exposed to BCL is associated with a modulation of the endocannabinoid system (ECS). To this aim, we used eight experimental groups of Sprague-Dawley rats, of which six were exposed to BCL for 24 hours. Following retinal function evaluation, retinas were quickly removed for biochemical and morphological analyses. Rats were either saffron-prefed or intravitreally injected with selective type-1 (CB_1_) or type-2 (CB_2_) cannabinoid receptor antagonists before BCL. Prefeeding and intravitreally injections were combined in two experimental groups before BCL. BCL exposure led to enhanced gene and protein expression of retinal CB_1_ and CB_2_ without affecting the other ECS elements. This effect of BCL on CB_1_ and CB_2_ was reversed by saffron treatment. Selective CB_1_ and CB_2_ antagonists reduced photoreceptor death, preserved morphology and visual function of retina, and mitigated the outer nuclear layer (ONL) damage due to BCL. Of interest, CB_2_-dependent neuroprotection was more pronounced than that conferred by CB_1_. These data suggest that BCL modulates only distinct ECS elements like CB_1_ and CB_2_, and that saffron and cannabinoid receptors could share the same mechanism in order to afford retinal protection.

## Introduction

Progressive degenerative diseases of retina, including age-related macular degeneration (AMD), diabetic retinopathy, retinitis pigmentosa, uveitis, retinal detachment as well as eye cancers (ocular melanoma and retinoblastoma), represent a large group of conditions that affect visual function in young and old people [2,3]. Photoreceptors are the major targets of many of these retinal diseases [4], and a sequence of events has been shown to lead to their malfunction and eventually to death, [5]. Recently, it has been shown that photoreceptor death can be reduced in several animal models of neurodegeneration, by using both neuroprotectants [6] and antioxidants [7], and remarkably saffron (see for ref [8]). Saffron (*Crocus sativus*) is a well-known spice largely used in traditional medicine [9,10]. Its efficacy in slowing down retinal degenerative processes in rats exposed to high intensity light has been recently documented [1]. In particular, orally administered saffron partially preserved both morphology and function in light damaged retina [1]. A pilot clinical trial conducted on AMD patients provided the first evidence of successful saffron treatment in therapy [11], the positive effects being maintained in time [12] and in patients carrying genetic mutation [13]. Multiple actions of saffron have been suggested, including modulation of gene expression in animal models of retinal degeneration [14]. The latter process activates rather complex pathways, with many receptors and diffusible molecules playing pivotal roles in disease progression and activation of neuroprotective mechanisms [15,16]. All of them represent, indeed, potential targets of neuroprotectants. In the last few years, a new family of lipid mediators, called endocannabinoids (eCBs), received attention as possible activators of retina protection mechanisms in an animal model of ganglion cell death induced by high-intraocular pressure [17]. eCBs, such as *N*-arachidonoylethanolamine (anandamide, AEA) and 2-arachidonoylglycerol (2-AG), represent major neurotransmitters/neuromodulators in neural cells [18]. They bind to type-1 (CB_1_) and type-2 (CB_2_) cannabinoid receptors [19] and to the transient receptor potential vanilloid type 1 (TRPV1) channels [20,21]. Several enzymes are involved in eCB metabolism: AEA is synthesized mainly by *N*-acyl-phosphatidylethanolamines-specific phospholipase D (NAPE-PLD), and is degradated by fatty acid amide hydrolase (FAAH); 2-AG is mainly synthesized by a *sn*-1-specific diacylglycerol lipase (DAGL) and is degraded by a specific monoacylglycerol lipase (MAGL) [22–24]. Altogether eCBs, their target receptors and metabolic enzymes form the so-called endocannabinoid system (ECS) [25]. Incidentally, rat retina has been already shown to possess several components of a functional ECS [17].

Against this background, here we sought to investigate whether bright continuous light (BCL) could modulate ECS in the rat retina, and whether saffron treatment could exert a neuroprotective effect by involving distinct elements of this signaling system.

## Materials and Methods

All experiments were conducted in accordance with ARVO Statement for the Use of Animals in Ophthalmic and Vision Research, authorization number 83/96-A of 29/11/1996 by the Ministry of Health, and were approved by the local Ethical Committee of University of L’Aquila. Animals were born and reared in dim cyclic light conditions (12 hours light, 12 hours dark) with an ambient light level of approximately 5 lux [1].

Data reported in this study were obtained from experiments carried out on 78 Sprague Dawley (SD) rats 3 months old, divided in eight groups as described below (in brackets are the same abbreviations used in the Results section):

1. Group: control SD rats (control), (n = 6);
2. Group: SD rats treated with stigmas of saffron for two weeks (saffron);
3. Group: SD rats exposed to BCL, 1000 lux for 24 h (light damage, LD);
4. Group: SD rats treated with stigmas of saffron for two weeks and exposed to BCL (saffron+LD);
5. Group: SD rats injected intravitreally with the selective CB_1_ antagonist SR141716A [26] in the right eye (SR1+LD) and with saline in the left eye, and then exposed to BCL;
6. Group: SD rats injected intravitreally with the selective CB_2_ antagonist SR 144528 [26] in the right eye (SR2+LD) and with saline in the left eye, and then exposed to BCL;
7. Group: SD rats pretreated with saffron for two weeks, injected with SR141716A in the right eye and then exposed to BCL (saffron+SR1+LD);
8. Group: SD rats pretreated with saffron for two weeks, injected with SR144528 in the right eye and then exposed to BCL (saffron+SR2+LD).

For each experimental group 12 animals were used; 6 were sacrified immediately after LD, while the other half (n = 6) seven days after light exposure.

### Diet supplementation

Albino rats were fed daily with a water suspension of 5 mg/kg stigmas as reported [1]. To avoid to use saffrons coming from different cultivars that may present different composition of the extract, in this paper we used only saffron derived from Hortus Novus (L’Aquila, Italy), whose chemical characteristics has been analytically determined (see for ref [27]).

### Light exposure

Animals were placed in individual plexiglass cages with food available on the floor, water in plastic bottles and dark adapted overnight. At 9 am they were exposed to BCL (1000 lux) for 24 hours, as reported [1]. For each experimental group, half of animals were immediately euthanized after LD, and the other half were allowed to recover for one week after BCL exposure. Retinas for biochemical analysis were immediately removed and frozen at –80°C.

### Intravitreal injections

Immediately before BCL, rats were anaesthetized by intraperitoneal injection of ketamine/xylazine (10 mg/100g – 1.2 mg/100g), were placed on the stereotactic microscope and a drop of local anesthetic (novocaine) was administered to each eye. 0.1 μM of SR141716A or SR144528 in 2 μl 0.9% NaCl were injected intravitreally in the right eye (of groups 5–8) using an Hamilton syringe with fixed needle (SYR 10 μl., ga 26s/51mm). Left eye was injected with 2 μl 0.9% NaCl alone, as a control. After the procedure the animals were located in post-operative cages and monitored until complete awakening. The binocular injection allowed the halving of the number of animals undergone to surgery since we did not included the control group (saline).

### Quantitative RT-PCR analysis

RNA was extracted from rat retinas by using the RNeasy extraction kit (Qiagen, Crawley, UK), as suggested by the manufacturer. Quantitative real time reverse transcriptase-polymerase chain reaction (qRT-PCR) assays were performed using the SuperScript III Platinum Two-Step qRT-PCR Kit (Invitrogen, Carlsbad, CA, USA). One μg total RNA was used to produce cDNA with 10 U/μL SuperScript III reverse transcriptase, in the presence of 2 U/μL RNaseOUT, 1.25 μM oligo(dT)20, 1.25 ng/μL random hexamers, 5mM MgCl_2_, 0.5 mM dNTP mix and DEPC-treated water. The reaction was performed by using the following qRT-PCR program: 25°C for 10 min, 42°C for 50 min, 85°C for 5 min; then, after addition of 0.1 U/μL of *E*. *coli* RNase H, the product was incubated at 37°C for 20 min. Target transcripts were amplified using an ABI PRISM 7700 sequence detector system (Applied Biosystems, Foster City, CA), with the following primers: NAPE-PLD F, 5’-TGTCCCGGGTTCCAAAGAGGAGC-3’, NAPE-PLD R, 5’-ACCATCAGCGTCGCGTGTCC- 3’; DAGL F 5’-ATTCTCTCCTTCCTCCTGC-3’, DAGL R 5’-ATTTGGGCTTGGTGCTTCG-3’; FAAH F 5’-ATGGAAGTCCTCCAAGAGC-3’, FAAH R 5’-TAGAGCTTTCAGGCATAGCG-3’; MAGL F 5’-ATGTTGAAGAGGCTGGACATGC-3’, MAGL R 5’-ATGCAGATTCCGGATTGGC-3’; CB_1_ F, 5’-TTCCACCGTAAAGACAGCCC-3’, CB_1_ R, 5’- TCCACATCAGGCAAAAGGCC-3’; CB_2_ F, 5’-TTGACCGATACCTATGTCTGTGC-3’, CB_2_ R, 5’-TGCTTTCCAGAGGACATACCC-3’; TRPV1 F 5’-ATTGAACGGCGGAACATGACG-3’, TRPV1 R 5’-ATCTCTTCCAGCTTCAGCG-3’; β-Actin F, 5’- ATCCTGACCCTGAAGTACCC-3’, β-Actin R, 5’- AAGGTCTCAAACATGATCTGG- 3’. Differences in threshold cycle (Ct) number were used to quantify the relative amount of PCR target in each tube. Relative expression of different gene transcripts was calculated by the ΔΔCt method, and was converted to relative expression ratio (2^−ΔΔCt^) for statistical analysis. β-Actin was used as housekeeping gene for quantification [28].

### Analysis of protein expression

Retinal lysates were obtained by sample homogenization in ice-cold lysis buffer (10 mM EDTA, 50 mM Tris-HCl (pH 7.4), 150 mM sodium chloride, 1% Triton-X-100, 2 mM phenylmethylsulfonylfluoride, 2 mM sodium orthovanadate, 10 mg ml^– 1^ leupeptin, and 2 mg ml^– 1^ aprotinin), and the amount of proteins was determined by the Bio-Rad Protein assay (Bio-Rad Laboratories, Hemel Hempstead, UK). Equal amounts of total extracts (30 μg of protein) were electrophoresed on 10% acrylamide gels and transferred to polyvinylidene fluoride membranes (Amersham Biosciences, Psicataway, NJ, USA). Membranes were saturated with a solution of 5% nonfat dry milk, then were incubated with anti-NAPE-PLD (1:100) (Cayman Chemicals, Ann Arbor, MI, USA; item n. 1035), anti-FAAH (1:500), anti-DAGL (1:1000) (Santa Cruz Biotechnology Inc., Santa Cruz, CA; sc-26427, sc-133307), anti-MAGL (1:200) anti-CB_1_ (1:250), anti-CB_2_ (1:250) (Cayman Chemicals, Ann Arbor, MI, USA, item n. 10035, n. 10006590, n. 101550), anti-TRPV1 (1:200) antibodies or with anti-β-actin (1:1000) antibody (Santa Cruz Biotechnology Inc., Santa Cruz, CA, sc-12498. Sc-1616). Then, they were incubated with specific horseradish peroxidase-conjugated (HRP) secondary antibodies diluted 1:2000 (Santa Cruz Biotechnology Inc., Santa Cruz, CA, USA). Detection was performed by using the West Dura Chemiluminescence System (Pierce, Rockford, IL, USA), and the intensity of the immunoreactive bands was quantified by densitometric analysis through the ImageJ software (NIH, Bethesda, MD, USA). The specificity of each antibody used was tested in rat as already reported [17,29,30].

In some experiments, protein expression of CB_1_ and CB_2_ was also determined by enzyme linked immunosorbent assay (ELISA), as reported [31]. Briefly, wells were coated with retinal lysates (20 μg/well) and were incubated for 1 h at room temperature with anti-CB_1_ or anti-CB_2_ polyclonal antibodies at the same dilutions used in Western blotting analysis. After rinsing three times with 5% BSA/PBS-Tween 20, 100 μl of HRP-conjugated secondary antibody (diluted 1:5000) was added and the ELISA plate was further incubated for 30 min at room temperature. HRP enzymatic activity was determined by the addition of 100 μL/well of tetramethylbenzidine (TMB) containing H_2_O_2_ (0.002%), and the absorbance was read on a Multiskan ELISA Microplate Reader (ThermoLabsystems, Bevery, MA, USA) at 450 nm. Results were expressed as a percentage of the control (100%).

### Morphology and immunohistochemistry

Animals were sacrificed immediately after LD, the eyes were enucleated, fixed, embedded, cryosectioned and immunostained. Sections were labelled for apoptotic cell death using the terminal deoxynucleotidyltransferase d-UTP nick end labeling (TUNEL) technique following protocols, as previously described [32]. Counts of TUNEL+ (apoptotic) cells in the outer nuclear layer ONL were made using a calibrated 20 x objective. Each section was scanned from the superior to inferior edge, and the number of TUNEL+ cells was recorded for each 400 μm length of the section. The total number of TUNEL+ cells for each experimental group was normalized respect to LD group.

Retinal sections were also immunolabeled for CB_1_ and CB_2_, removing non-specific binding with 0.75% horse serum. Sections were incubated with rabbit anti-CB_1_ (overnight at 4°C) or anti-CB_2_ (3 days at 4°C) polyclonal antibodies diluted 1:200. For immunohistochemistry of CB_2_ a different antibody was used abcam ab3561), compared to Western Blotting analysis, because it is designed for use with frozen tissues sections. Secondary antibody was anti-rabbit IgG conjugated to fluorescent dye (Alexa Fluor 594 or 488; Life Technology) diluted 1:200 and incubated at 37°C for 2 hours. At the end of the procedure the images were taken by confocal microscope (Nikon 80i), as reported [1].

To evaluate the entity of the damage in the superior retina, the extension of the “hot spot” was measured. This analysis was performed in retinal sections one week after BCL. Sections were labelled with the DNA-specific dye bisbenzimide (Calbiochem, La Jolla, CA), by incubating them for 2 min in a 1:10.000 solution in 0.1 M PBS. Images were taken by confocal microscope (Nikon 80i).

### Electrophysiological recordings

To evaluate visual function, electroretinogram (fERG) in response to flashes of increasing luminance was recorded one week after BCL. Albino rats were dark adapted for a 12 hour period overnight and electroretinograms were recorded in a completely darkened room [33]. Briefly, animals were anaesthetized by an intraperitoneal injection of Ketamine/Xylazine (10 mg/100g–1.2 mg/100g) and mounted in a stereotaxic apparatus and positioned inside the opening of the Ganzfeld dome (Biomedica Mangoni, Pisa, Italy). The body temperature was maintained at 37.5°C with a heating pad controlled by a rectal temperature probe. Corneas were anesthetized with a drop of novocaine, and pupils were dilated with 1% tropicamide. This electronic flash unit generated flashes of a range of intensities from 0.001–100 cd/m^2^. Responses were recorded over 300 ms plus 25 ms of pre-trial baseline, amplified differentially, bandpass filtered at 0.3 to 300 Hz, digitized at 0.25- to 0.3-ms intervals by a LabVIEW 8.2 personal computer interface (National Instruments, Milan, Italy). The amplitude of the b-wave was measured from the most negative point of the average trace to the highest positive point. At the end of the recording session, animals were sacrificed, the eyes removed and retinas were used for retinal histology.

### Statistical Analysis

Data are reported as means ± S.E.M of at least six independent experiments, each performed in duplicate. Data were analysed by the Prism 5 program (GraphPad Software, La Jolla, CA), using one-way analysis of variance (ANOVA) followed by Tukey test or Bonferroni *post hoc* analysis. A level of p<0.05 was considered statistically significant.

## Results

### Effect of BCL on expression of ECS genes and proteins

**Fig 1:**
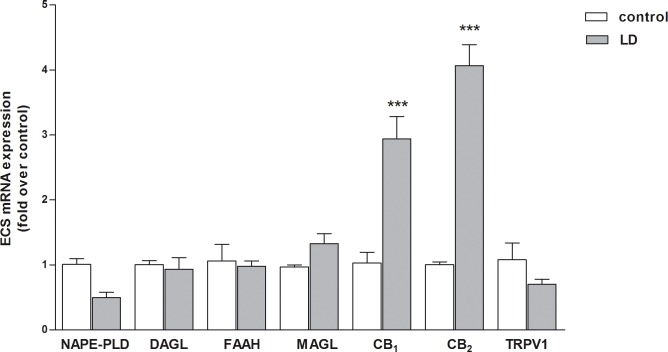
Effect of BCL on ECS mRNA expression. qRT-PCR analysis of main components of ECS (NAPE-PLD, DAGL, FAAH, MAGL, CB_***1***_, CB_***2***_ and TRPV1) in the retinas from untreated controls and rats exposed to bright continuous light (LD). Data were expressed as means ± SEM (n = 6), and were analyzed by one-way analysis of variance (ANOVA) followed by Bonferroni post hoc analysis. ***p<0.001 vs control.

In the first set of experiments, the effects of retinal damage induced by exposure to BCL were assayed on ECS expression, by means of qRT-PCR and Western blotting analyses. These procedures were carried out on 12 animals belonging to 1° and 3° group (control and LD without recovery). The results of qRT-PCR experiments on gene expression of the main components of ECS in retina from LD rat are shown in Fig 1. Only CB_1_ and CB_2_ mRNA levels increased in the retinas of LD rats with respect to controls, by ~3-fold and ~4-fold respectively. Instead, none of the other ECS elements tested (*i*.*e*., NAPE-PLD, DAGL, FAAH, MAGL and TRPV1) was affected (Fig 1).

**Fig 2:**
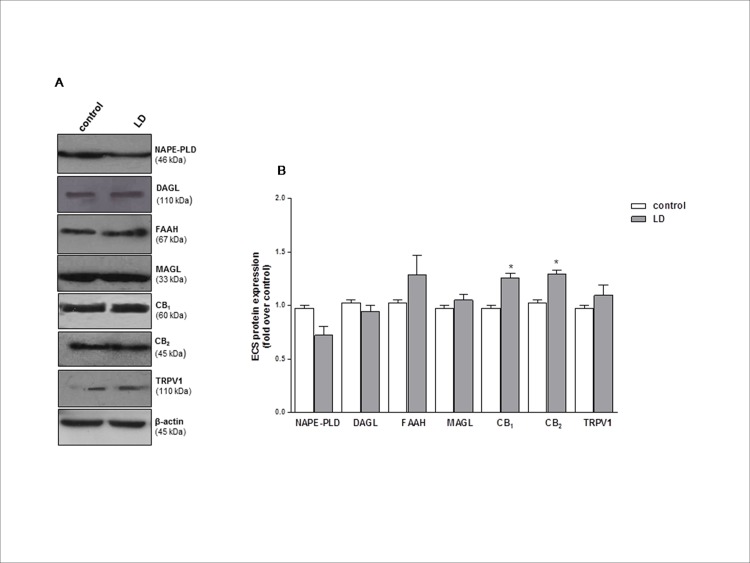
Effect of BCL on ECS protein expression. (A) Representative Western blot of main components of ECS (NAPE-PLD, DAGL, FAAH, MAGL, CB_***1***_, CB_***2***_ and TRPV1) in the retinas from controls and rats exposed to bright continuous light (LD). Panel B shows densitometry of ECS components expression normalised for its own control. Data were expressed as means ± SEM (n = 6), and were analyzed by one-way analysis of variance (ANOVA) followed by Bonferroni post hoc analysis.*p<0.05 vs control.

In keeping with these mRNA data, Western blotting (Fig 2) showed a significant increase (p<0.05) only in the expression of CB_1_ and CB_2_ proteins in LD rats with respect to controls.

### Effect of saffron on BCL-induced expression of CB_1_ and CB_2_

**Fig 3:**
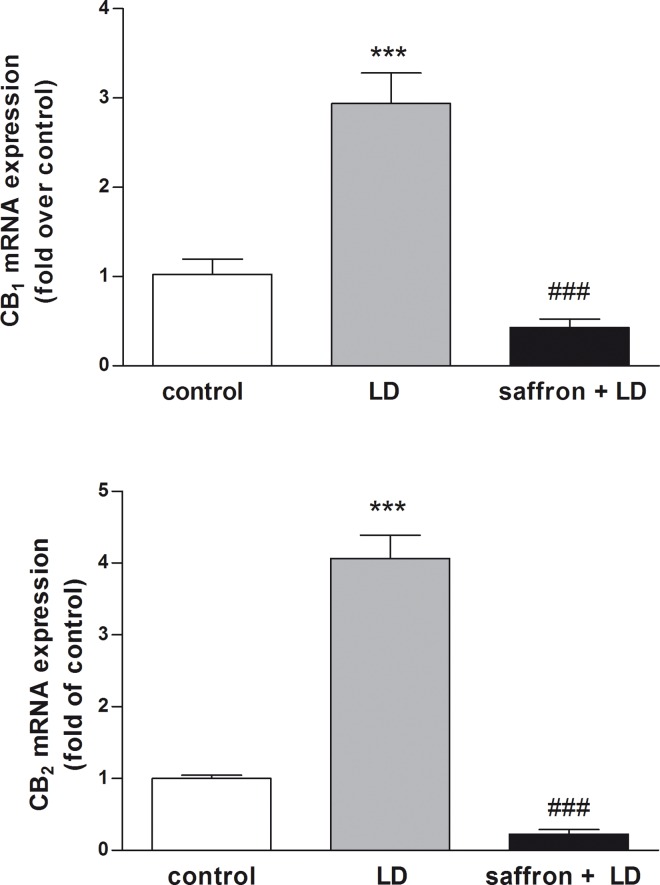
CB_1_ and CB_2_ mRNA levels following saffron treatment and BCL exposure. qRT-PCR analysis of CB_***1***_ and CB_***2***_ in the retinas from untreated controls, rats exposed to bright continuous light alone (LD), or in combination with saffron (saffron + LD). Data were expressed as means ± SEM (n = 6), and were analyzed by one-way analysis of variance (ANOVA) followed by Bonferroni post hoc analysis. ***p<0.001, ****p<0.0001 vs control, ###p<0.001, ####p<0.0001 vs LD.

In order to investigate the involvement of CB_1_ and CB_2_ in the neuroprotective effect of saffron against BCL, further analyses were performed in retinas from rats prefed with saffron. Interestingly, after two weeks of treatment with saffron stigmas and after exposure to BCL (without recovery), mRNA levels of CB_1_ and CB_2_ decreased significantly (p<0.001 for CB_1_; p<0.0001 for CB_2_), and returned to controls (Fig 3).

**Fig 4:**
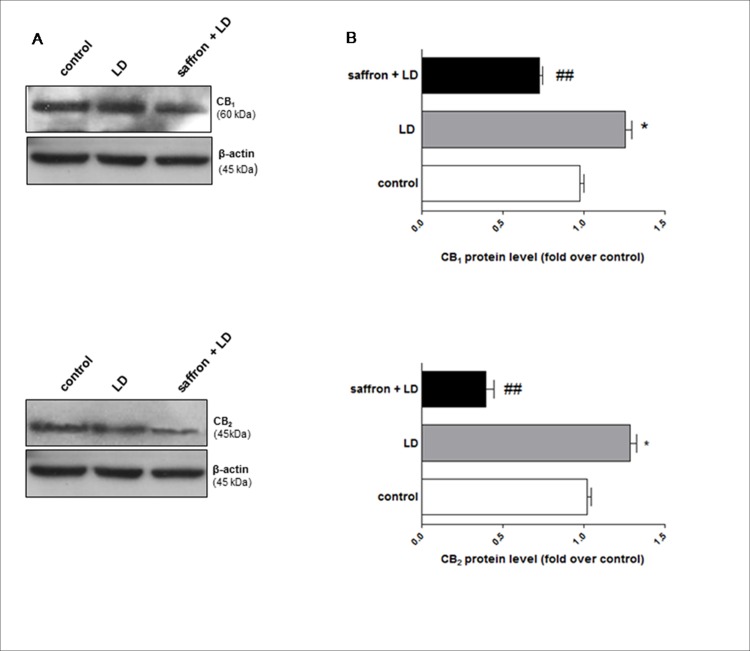
CB_1_ and CB_2_ protein levels following saffron treatment and BCL exposure. (A) Representative Western blot of CB_***1***_ and CB_***2***_ in the retinas from controls, rats exposed to bright continuous light alone (LD), or in combination with saffron (saffron + LD). Panel B shows densitometry of CB_***1***_ and CB_***2***_ expression normalised for its own control. Data were expressed as means ± SEM (n = 6), and were analyzed by one-way analysis of variance (ANOVA) followed by Bonferroni post hoc analysis.*p<0.05 vs control, ##p<0.01 vs LD.

Similarly, at protein level a significant reduction of CB_1_ and CB_2_ (p< 0.01) expression was observed following saffron treatment (saffron+LD) with respect to LD group (Fig 4). Additional ELISA assays (Table 1) confirmed the increase of CB_1_ and CB_2_ (p<0.001 for CB_1_; p<0.01 for CB_2_) protein expression in LD rats compared to controls, as well as their reduction upon saffron treatment (saffron+LD). Instead, no statistically significant differences were found between saffron and control groups (data not shown). In particular, the effect of saffron on CB_2_ transcription and translation was larger than that on CB_1_, suggesting a stronger engagement of this receptor in retinal protection by saffron. These analysis were performed on 18 animals (groups 1°, 3° and 4°).

**Table 1:** Effect of BCL exposure on CB_1_ and CB_2_ protein content after saffron treatment.

Experimental groups	CB_1_	CB_2_
**control**	100 ± 1	100 ± 6
**LD**	180 ± 5***	140 ± 6**
**saffron + LD**	124 ± 7^##^	42 ± 2^####^

Values of ELISA tests were expressed as percentage of control (100% = 1.00 ± 0.01 A_450_ units for CB_1_; 100% = 1.03 ± 0.01 A_450_ units for CB_2_).

**p<0.01 and

***p<0.001 *vs* control

^##^p<0.01 and

^####^p<0.0001 *vs* LD.

### Localization of CB_1_ and CB_2_

**Fig 5:**
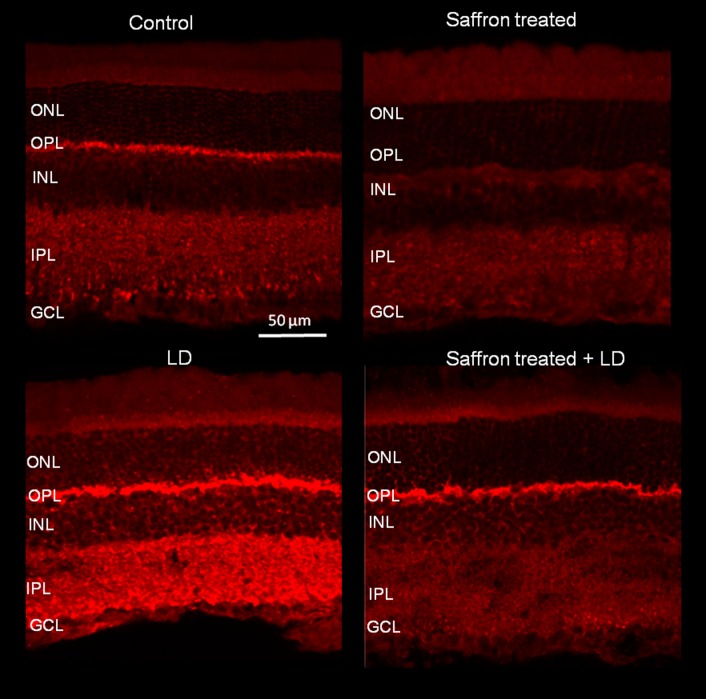
CB_1_ immunolabelling following saffron treatment and BCL exposure. Localization of CB_***1***_ in retinal sections. Images are representative of the same retinal region in the four tested groups: control, saffron, LD, saffron+LD. Scale bar: 50 μm. ONL (outer nuclear layer), OPL (outer plexiform layer), INL (inner nuclear layer), IPL (inner plexiform layer), GCL (ganglion cell layer).

**Fig 6:**
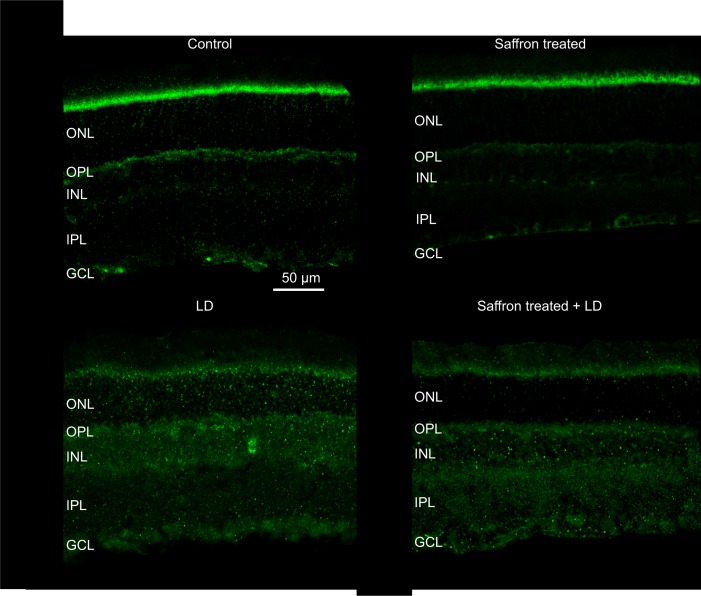
CB_2_ immunolabelling following saffron treatment and BCL exposure. Localization of CB_***2***_ in retinal sections. Images are representative of the same retinal region in the four tested groups: control, saffron, LD, saffron+LD. Scale bar 50 μm.

In order to confirm the modulation of CB_1_ and CB_2_ by saffron treatment, their retinal localization was evaluated through immunohistochemistry technique in 24 animals (1°, 2°, 3° and 4° group) (Figs 5 and 6). Our data demonstrate that CB_1_ was localized in both outer and inner plexiform layers (OPL and IPL) of all experimental groups. In particular, axon terminals of bipolar cells were labelled in the IPL of controls (Fig 5), in keeping with a previous report [34]. After exposure to BCL (without recovery), CB_1_ immunoreactivity increased without any change in receptor localization (LD group in Fig 5). Saffron treatment reduced CB_1_ expression after exposure to LD (saffron+LD group). The immunofluorescence intensity of CB_1_ in saffron group appears reduced respect to control, although quantitative analysis of protein level did not show any statistical difference between the two groups (Fig 5). Similar effect was observed also for CB_2_ immunolocalization. Indeed, in all experimental groups CB_2_ was mainly localized in the inner segment of photoreceptors and also in the inner retina (inner nuclear layer and ganglion cell layer) (Fig 6), extending previous studies [35]. After exposure to BCL (LD group in Fig 6), CB_2_ immunoreactivity increased in the inner retinal layers and was reduced by saffron treatment (saffron and saffron+LD groups in Fig 6).

### Quantitative analysis of apoptotic cells

**Fig 7:**
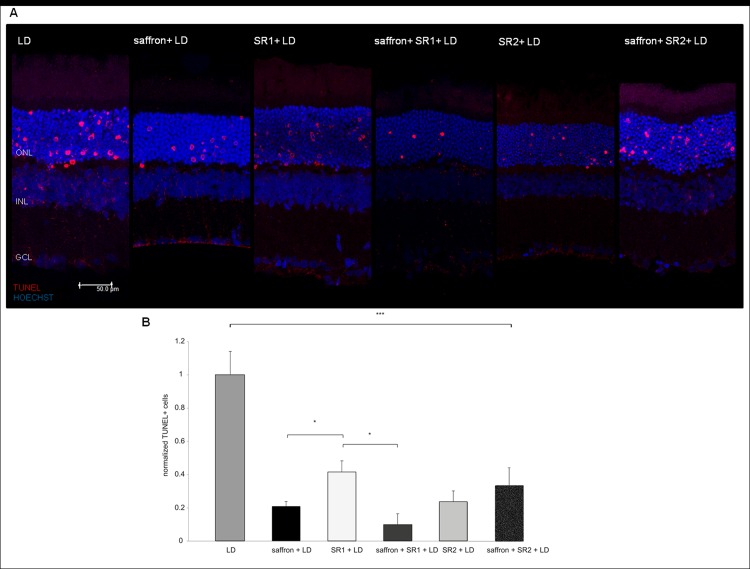
Quantitative analisys of TUNEL+ cells. Fig 7A: representative images acquired by confocal microscopy in the same region (“hot spot”) showing the apoptotic cells after exposure to bright light in the six experimental conditions examined. The quantitative analysis of the dying cells in outer nuclear layer (ONL) is reports in the graph (panel B). Dying photoreceptors are counted from superior to inferior edge in all experimental conditions normalized respect to LD group. Data were expressed as means ± SEM (n = 6), and were analyzed by one-way analysis of variance (ANOVA) followed by Tukey-test post hoc analysis. ***p<0.001 all experimental groups vs LD, *p<0,05 SR1+LD vs saffron+LD and vs saffron+SR1+LD.

It has already demonstrated that exposure to BCL (24 hours at 1000 lux) induces photoreceptor death; the maximum level of apoptotic cells is evident immediately after damaging light particularly in the superior retina, an area known as “hot spot” [36,37,8]. This area is represented in Fig 7A where dying photoreceptors (red dots) are evident in the ONL. In Fig 7B we report the normalized TUNEL+ (apoptotic) cells, showing a highly significant reduction of apoptotic cells in all experimental groups respect to LD group. Saffron treatment significantly reduced the number of dying neurons in line with previous data [1]. Interestingly, intravitreal pre-treatment with CB_1_ (SR1+LD group) or CB_2_ (SR2+LD group) antagonists also reduced neuronal death, though in a slightly less efficient manner than saffron. Supplementation with the latter substance reduced further the number of TUNEL+ cells only in rats pre-treated with CB_1_ antagonist (saffron+SR1+LD group), suggesting that saffron and CB_1_ may share the same transduction pathways. This conclusion was supported by the ELISA assay (Table 1).

### Retinal function

**Fig 8:**
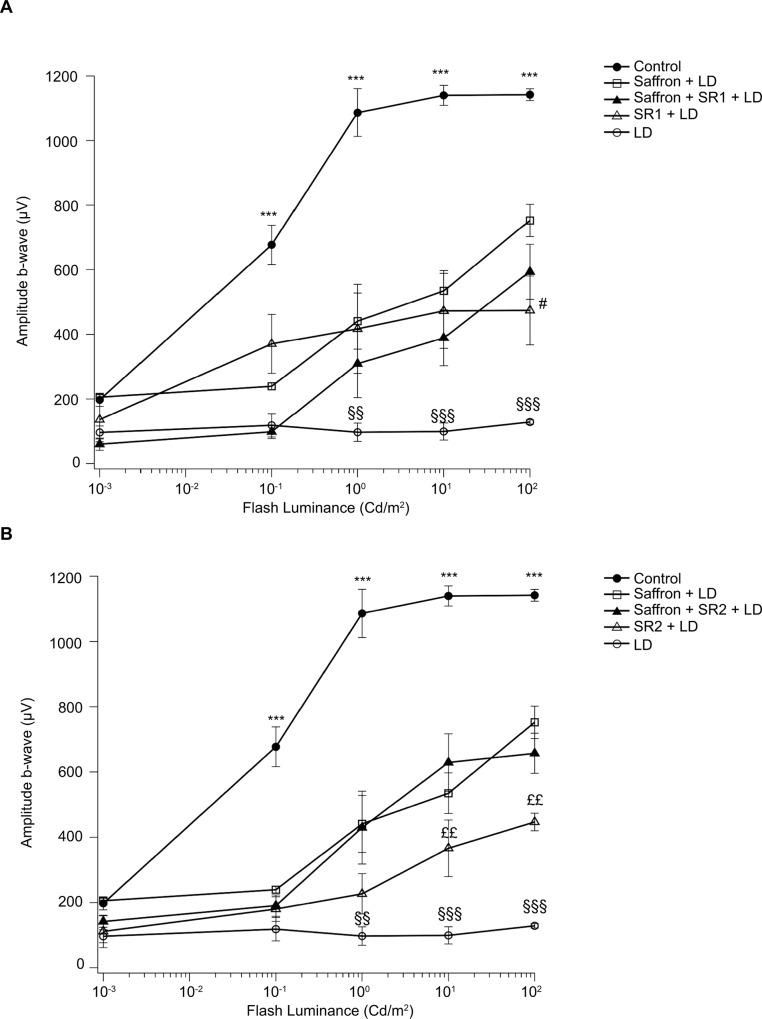
fERG b-wave amplitude (μV) as a function of flash luminance (cd/m^2^). In both panels A and B are reported the same data for control, LD and saffron+LD. Panel A: CB_***1***_ receptors antagonist [SR141716A (SR1)] with or without saffron, panel B: CB_***2***_ receptor antagonist [SR144528 (SR2)], with or without saffron. Data were expressed as means ± SEM (n = 6). # p<0.05 SR1+LD vs saffron+LD; ££p<0,01 SR2+LD vs saffron+LD; §§ p<0.01 and §§§p<0.001 LD vs saffron+LD, SR1+LD, saffron+SR1+LD, SR2+LD, saffron+SR2+LD; ***p<0.001 control vs all experimental groups.

Retinal function was evaluated one week after exposure to BCL in all experimental groups, by recording fERG under dark-adapted conditions (Fig 8). The amplitude of the b-wave was strongly reduced by LD, an effect counteracted by saffron treatment (saffron+LD group), as already reported [1]. Moreover b-wave amplitude, recorded in rats intravitreally pre-treated with SR1 or SR2 with or without saffron supplementation, was improved respect to that of LD group (Fig 8A and 8B). Indeed, Fig 8A shows that b-wave amplitude recorded after inactivation of CB_1_ (SR1+LD group) was super imposable on that of the saffron + LD group, yet only at a low intensity light stimulus (0.001–10 cd/m^2^). At higher intensity (100 cd/m^2^), the reduction of fERG response was still significant, but the response of the SR1 + LD group differed from that of the saffron + LD group (Fig 8A). Co-administration of saffron and SR141716A (saffron+SR1+LD group) did not further improve b-wave amplitude (Fig 8A). The same analysis of fERG response was performed after inactivation of CB_2_ by SR144528 (SR2+LD group) with or without saffron treatment (Fig 8B). Upon SR2 treatment, b-wave amplitude increased compared to LD alone, especially at high intensity light flashes (100 cd/m^2^); (Fig 8B). A combination of saffron and SR2 (saffron+SR2+LD group) yielded a fERG response super imposable on that of the saffron + LD group (at least up to 100 cd/ m^2^ Fig 8B).

### Morphological analysis

**Fig 9:**
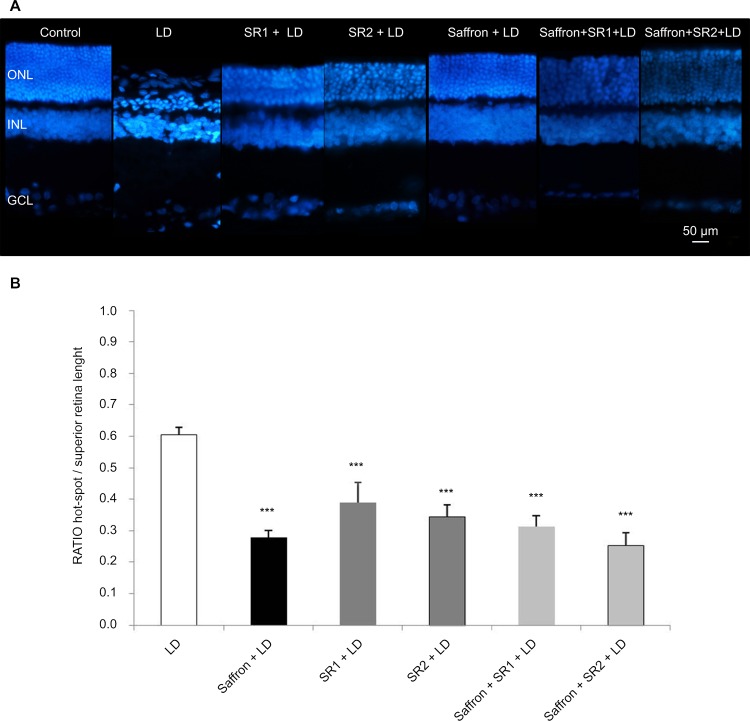
Bisbenzimide labelling and quantitative analysis of hot spot extension. Panel A: Representative images labelled with a nuclear staining (bisbenzimide) in a dorsal position one millimeter from optic disc (“hot spot”) in LD, saffron+LD, SR1+LD, saffron+SR1+LD, SR2+LD, saffron+SR2+LD, one week after light damage (LD). Scale bar, 50 μm. Panel B: Ratio of hot spot/superior retinal length. Data were expressed as means ± SEM (n = 6). *** p<0.001 vs LD.

The superior retina, from dorsal edge to optic nerve, was analyzed in order to evaluate the extension of degeneration induced by light exposure under all experimental conditions (Fig 9). Representative images of nuclear staining with bisbenzimide showed a specific dorsal area of ONL, called “hot spot” (Fig 9A). Light exposure induced a maximal damage to this region (Fig 9A), in keeping with a previous report [38]. Comparing ONL morphology across all groups, photoreceptor layer appeared well-preserved compared to the LD group; in particular, retinal morphology of the saffron group was very close to that of controls (Fig 9A). To better assess the rate of photoreceptor survival, the extension of the hot spot area was measured with respect to the entire length of the superior retina (Fig 9B). The least damaged area was observed in the saffron + LD group, but also SR141716A and SR144528 decreased hot spot damage, suggesting that selective blockage of CB_1_ and CB_2_ can protect photoreceptors against environmental stress (Fig 9B). Again, saffron in combination with CB_1_ (saffron+SR1+LD group) or CB_2_ antagonists (saffron+SR2+LD group) did not induce any additional protective effect (Fig 9B).

Regarding the intravitreal injections in both eyes (vehicle and antagonist respectively), even though a cross-talk was present between both eyes, the different effect between the two allowed us to conclude that antagonist administration protects neuroretina independently of the mechanical damage (that was the same in both eyes). In addition, results obtained after saline injections have demonstrated the absence of a systemic effect of the antagonists (SR1, SR2). Incidentally, we chose to skip data of the contralateral retinae in the plots, because they did not add any further information.

## Discussion

The presence of ECS in retina is well-documented in numerous species from fishes to primates [15,35,39,40] although it has been recently pointed out that “the biological functions of eCBs, TRPV1 and their interactions across retinal circuits remain almost entirely unknown” [41]. The evidence for 2-AG metabolic enzymes in rat retina is demonstrated here for the first time at the gene level, extending previous data on localization of DAGL and MAGL proteins during postnatal development of the rat retina [42]. Present data also extend previous evidence on the neuroprotective role of saffron in retinal damage [1], and suggest an unprecedented engagement of eCB signaling in this process. Indeed, eCBs have been reported to regulate photoreception and neurotransmission in the retina, and to have effects on intraocular pressure and ocular blood vessels [15,17,41,43] as well as neuroprotective effects against retinal neurotoxicity [15,44]. In line with this, in a previous study we demonstrated that anandamide is neuroprotective against retinal ganglion cell death induced by high intraocular pressure, *via* a CB_1_-dependent pathway [17]. Here, we provide the first evidence that BCL selectively affects ECS gene and protein expression in retina, where only CB_1_ and CB_2_ levels were increased. Instead, the other major components of retinal ECS were not modulated by BCL, including TRPV1 that plays a role in retinal death induced during IOP-related disease [17,41]. Apparently, saffron *per se* has little effect on retinal gene expression, but when administered before LD it does modulate the large changes in gene expression induced by this treatment [14]. In keeping with this notion, here we demonstrate that saffron down-regulates gene and protein expression of CB_1_ and CB_2_ in an animal model of retinal degeneration induced by light exposure. Consistently, we document that selective blockage of both CB_1_ and CB_2_ is able to reduce LD-induced photoreceptor death, thus preserving morphology and visual function, suggesting that these receptors are involved in neurodegenerative processes and are negatively modulated by saffron. Interestingly, literature data indicate that CB_2_ might be implicated in rod and cone sensitivity and light adaptation (see for ref. [35]). Consistently, our data suggest a major involvement of CB_2_ compared to CB_1_ in protecting photoreceptors from LD. Altogether our results indicate the possibility that the neuroprotective effects of saffron might impinge upon CB_1_/CB_2_ –dependent signal transduction pathways. Here, we found an increased amplitude in the b-wave of rats with retinal damage treated with CB_1_/CB_2_ antagonists but interestingly the b-wave amplitude in animal prefer with saffron or double treated with saffron and CB_1_ or CB_2_ antagonists are quite similar. The major difference happens to be at high luminance and always in favour of saffron only. In addition, accumulating evidence shows that CB_1_/CB_2_ levels are elevated in pathological retinal conditions sometime associated to oxidative stress [15,45]. Also in this study we observed such an increase after retinal damage induced by exposure to BCL, that often results in retina degeneration. Saffron is also endowed with a potent antioxidant activity, that has been attributed primarily to its crocins constituent (see for ref[8]). In line with this, reduction of inflammation due to the downregulation of chemokine CCL2 by saffron [14], and remarkably also by CB_1_/CB_2_ antagonists [46], could be a common pattern of response against retinal damage. Indeed, when the retina is damaged by bright light, a variety of pathways are activated, including an upregulation of Chemokin (C-C motif) Ligand 2 (CCL2) which recruits macrophages to scavenge retinal debris. Unsurprisingly, homozygous deletion of CCL2 results in a mouse phenotype similar to human AMD [47]. In general, CCL2 is implicated in inflammatory cell migration into inflamed tissues and nociception, processes that have been both related also to CB_2_-dependent signaling [48–50]. In this context, it should be recalled that CB_2_ plays a key-role in chemokine production and release by immune cells, i.e. microglia, thus regulating several inflammatory processes [51,52]. Retinal neuro-inflammation is strictly related to the activation of microglia (see for ref [53]), that in physiological condition maintain homeostasis in the retina also controlling synaptic activity in a continuous cross-talk with other retinal neurons. It can be suggested a direct control on CB receptors widely expressed across the retina and whose activation regulates calcium and potassium current. In conclusion, it might be that one of the neuroprotective pathways activated by saffron includes the activation of endocannabinoid system. Detailed analysis of a variety of possible neuroprotective ways of action is under investigation. Overall, topical CB_1_ and CB_2_ antagonists, in combination with saffron supplement in the diet, might be a potential novel treatment to cope with retinal neurodegenerative processes.
